# 3-Hydroxytyrosol Promotes Angiogenesis In Vitro by Stimulating Endothelial Cell Migration

**DOI:** 10.3390/ijms21103657

**Published:** 2020-05-22

**Authors:** Mario Abate, Simona Pisanti, Mariella Caputo, Marianna Citro, Carmine Vecchione, Rosanna Martinelli

**Affiliations:** 1 Department of Medicine, Surgery and Dentistry “Scuola Medica Salernitana”, University of Salerno, Via Salvador Allende, Baronissi, 84081 Salerno, Italy; mabate@unisa.it (M.A.); spisanti@unisa.it (S.P.); macaputo@unisa.it (M.C.); mcitro@unisa.it (M.C.); cvecchione@unisa.it (C.V.); 2 Vascular Pathophysiology Unit, IRCCS Neuromed, Via Atinense, Pozzilli, 86077 Isernia, Italy

**Keywords:** 3-Hydroxytyrosol, angiogenesis, cellular migration, endothelial cells

## Abstract

Cardiovascular diseases, followed by strokes, represent the leading cause of mortality worldwide. Despite its success in preventing cardiovascular diseases, the therapeutic potential of 3-Hydroxytyrosol (HT) for treating ischemic diseases is yet to be investigated in detail, especially with regard to ischemic heart disease, which is a major challenge for humans. We assessed that low concentrations (1–5 µM) of HT, generally achieved after the ingestion of olive oil, stimulate endothelial cells migration and angiogenesis in an in vitro model. At early time points (1–6 h), HT induces the expression of different proteins such as proto-oncogene tyrosine-protein kinase Src (Src), rho-associated protein kinase (ROCK) and matrix metalloproteinase-2 (MMP-2) protein influencing cell adhesion, cytoskeletal dynamics and cell migration. We observed that at the same time, HT induces prominent vascular formation in the tube formation assay, accompanied by an increase in the expression of the vascular endothelial growth factor receptor (VEGF-R2) and PI3K-Akt-eNOS protein pathways, which are recognized for their central role in angiogenesis. Therefore, in addition to the proven capability of HT to regulate reactive oxygen species (ROS) levels, through both direct scavenging properties and indirect antioxidant efficacy, our results revealed that HT promotes angiogenesis, arguing in favor of great pharma-nutritional potential in ischemic injuries.

## 1. Introduction

Cardiovascular diseases are a group of different disorders affecting heart and blood vessels—primarily arteries supplying blood to the heart, brain and other organs—that lead to acute complications responsible for ischemic conditions. Diseases of the cardiovascular system are quickly increasing worldwide. Recent studies predict that, in the next few years, cardiovascular diseases will exceed infectious diseases, and millions of people will be affected by morbidity and mortality [1].

To prevent tissue death after arterial occlusion, it is necessary to restore blood flow to the ischemic organ, but despite significant progress in the medical and surgical fields, preventive and therapeutic strategies are still lacking [1].

It is well recognized that the Mediterranean diet, which is characterized by an abundant intake of vegetables, fruit, legumes, cereals, fish, etc., is associated with increased longevity and improved cognitive function, and has proven cardio-protective action [2]. In addition, it is associated with a reported lower incidence of chronic degenerative diseases, as well as anti-tumor and anti-inflammatory properties [3]. Thus, the Mediterranean alimentary pattern displays beneficial effects in healthy people, and also represents a good basis for the prevention of numerous diseases and protection against overall morbidity and mortality.

Indeed, the polyphenols contained in olive oil, at the basis of Mediterranean diet pyramid, have well-recognized pharma-nutritional properties responsible for protective effects against cardiovascular diseases [4]. It is also known that 3, 4-dihydroxyphenylethanol (3, 4-DHPEA), or 3-Hydroxytyrosol (HT), the main phenolic component in high-quality extra virgin olive oil, which originates from the hydrolysis of oleuropein during olive ripening, exerts, among others, an antioxidant, anti-inflammatory, cardioprotective, neuroprotective, anti-platelet aggregation and anti-atherogenic action, both in vitro and in relevant animal models of cardiovascular pathologies [5,6].

Thanks to its antioxidant properties, HT prevents the oxidation of low-density lipoproteins (LDL), thus decreasing the formation of atherosclerotic plaques and reducing plasma levels of total cholesterol and lipids, along with blood pressure values [7,8]. HT shows an antithrombotic and anti-inflammatory activity preventing platelet aggregation through the inhibition of cAMP- (cyclic adenosine monophosphate) and cGMP- (cyclic guanosine monophosphate) phosphodiesterase (PDE), and is capable of inhibiting cyclooxygenase (COX) expression, thus reducing the production of thromboxane B2 (TxB2) and thromboxane A2 (TxA2) [9,10,11].

Recent studies show that HT is able to reduce cardiovascular risk in the early stages of atherosclerosis, decreasing the secretion of several adhesion molecules such as E-selectin, P-selectin, intercellular adhesion molecule 1 (ICAM-1) and vascular cell adhesion molecule 1 (VCAM-1) in human aortic endothelial cells (HAEC) [10,11]. Furthermore, it is well-recognized that HT can be used as an adjuvant molecule in anti-cancer therapies that cause an increased cardiovascular risk, as it improves cardiac disorders generated by ROS imbalance production and mitochondria damage, also facilitating an improvement in the anti-tumor response and fewer adverse effects associated with the treatment [12,13,14].

At the same time, no undesirable effects have been demonstrated, even at high doses, configuring the safety profile of HT as excellent [15,16]. This is the main reason why the European Food Safety Authority (EFSA) recommends a daily intake of at least 5 mg of HT, through the assumption of extra virgin olive oil [17,18].

Despite the preventive activity of HT against cardiovascular diseases, its direct effect on vessels’ healing and biology and on the molecular mechanisms involved have not yet been investigated. Its wide range of biological properties has been mainly ascribed to its strong antioxidant activity [19]. The vascular endothelium is implicated in cardiovascular diseases and strokes. To date, we know that a poor vascularization surrounding the ischemic zone can be the cause of a failure of recovery from ischemia. At the same time, the correct proliferation, migration and formation of novel endothelial cell tubes in the ischemic area can play a critical role in promoting angiogenesis for the treatment of cardiovascular diseases [20].

For this reason, in the present study, we have investigated for the first time the effect of HT on neovascularization. We have evaluated if HT can promote endothelial cell migration in vitro, influencing cell adhesion, cytoskeletal dynamics, signal transduction and the subsequent formation and maturity of new capillary tubes, thus finalizing neovascularization.

## 2. Results

### 2.1. Evaluation of HT Effect on Human Umbilical Vascular Endothelial Cells

**Figure 1:**
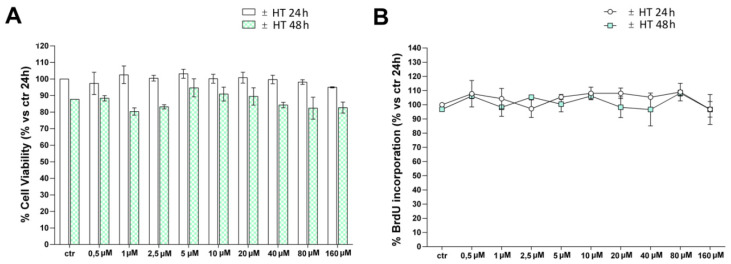
Evaluation of HT effect in HUVECs. HUVECs were cultured for 24 h or 48 h with different concentrations of HT (0–160 µM), before MTT assay (**A**) or BrdU incorporation (**B**). Results are expressed as mean (±SD), and are representative of four independent experiments carried out in triplicate. Data are reported as percentage vs. control (untreated cells at 24 h) (2-way ANOVA).

In order to study the effects of 3-Hydroxytyrosol (HT) on angiogenesis, we used a well-characterized cell line of human umbilical vascular endothelial cells (HUVEC). Cells were cultured with increasing concentrations of HT (0–160 µM) for 24 h and 48 h. It is important to note that HT did not affect cell viability even at the highest doses, as shown by MTT (3-(4, 5-dimethylthiazolyl-2)-2, 5-diphenyltetrazolium bromide) assay (Figure 1A) and cell proliferation, assessed by BrdU incorporation (Figure 1B). Subsequently, we have investigated the role of HT in neovascularization. Since high concentrations of HT cannot be achieved after the natural ingestion of olive oil, as polyphenols bioavailability is in the range of 0.1–1 μM, and since the time required for complete HT elimination is approximately 6 h [15,21], we conducted all the other experiments at early time points (within 6 h) and at low concentrations of 1–5 µM.

### 2.2. Improvement of the Migratory Capacity of Endothelial Cells Exposed to HT

**Figure 2:**
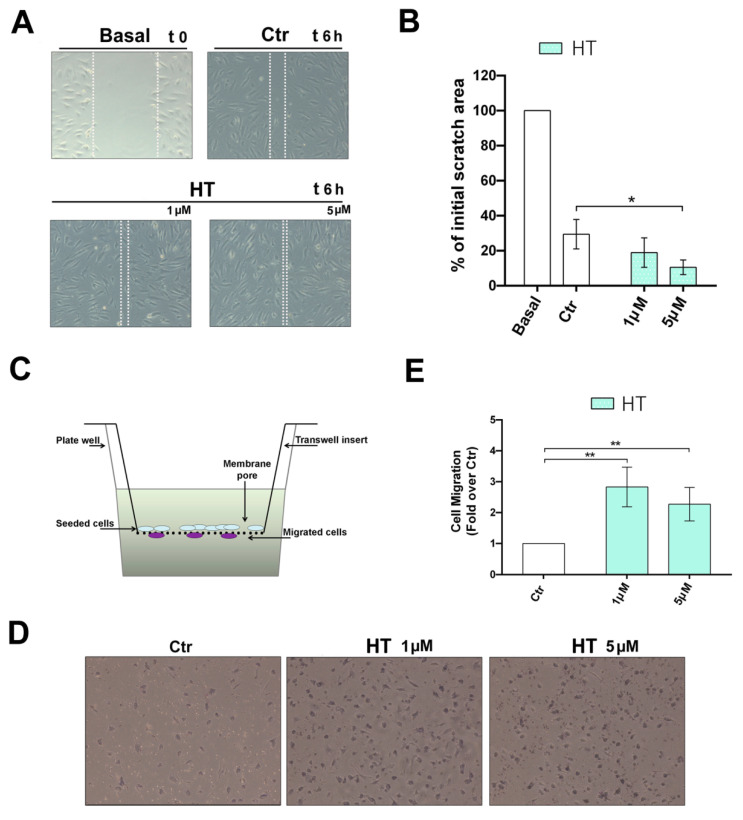
Improvement of the migratory capacity of HUVEC cells exposed to HT. (**A**) Wound healing assay were carried out in HUVECs treated for 6 h with HT at the indicated concentrations (1–5 µM) in complete medium. Light microscope images are representative of three independent experiments. Dotted white lines indicate the wounded area from the initial scratch. Magnification × 100; (**B**) Histograms correspond to the mean scratch area obtained in HUVEC cultures, and are expressed as a percentage with respect to the initial area. The measurement was carried out in three different experiments. Results are shown as mean (±SD) (2-way ANOVA, * *p* < 0.05). (**C**) Cell migration was determined in the Boyden chamber system after seeding HUVECs in the upper insert and treatment with HT. (**D**) Cells that migrated beneath the membrane were fixed and stained and representative light microscope images of three independent experiments are shown (10 × magnification). (**E**) The effects of HT on cell migration, at the indicated concentrations, were observed after overnight incubation. Results, reported as folds over the control, are shown as mean (±SD) (2-way ANOVA, ** *p* < 0.01).

Endothelial cell migration is essential for the formation and stabilization of new microvessels during the angiogenic process. It is directionally regulated and involves the degradation of the extracellular matrix to enable the progression of the migrating cells [22]. For this reason, in order to assess a potential effect of HT on the migratory function of HUVECs, we performed a scratch wound assay on a monolayer of confluent cells. The closure of the wounded area was monitored over this time. After 6 h of treatment with HT, we observed an enhancement of wound healing at both doses tested (1 µM and 5 µM (* *p* < 0.05)), as shown in Figure 2A,B. Moreover, to confirm whether HT is able to exert a pro-migratory chemotactic directional effect on endothelial cells, we stimulated HUVECs in a Boyden chamber system, as illustrated in Figure 2C. By counting the number of cells that migrated beneath the membrane through a proangiogenic stimulus, represented by the growth media and complete with all the angiogenic growth factors (Figure 2C,D), we observed that HT stimulated HUVEC migration at 1 µM and 5 µM (** *p* < 0.01), as shown in Figure 2E. These results confirm the stimulatory activity of HT on HUVEC migration.

### 2.3. HT Induces the Expression of Migration-Linked Proteins

**Figure 3:**
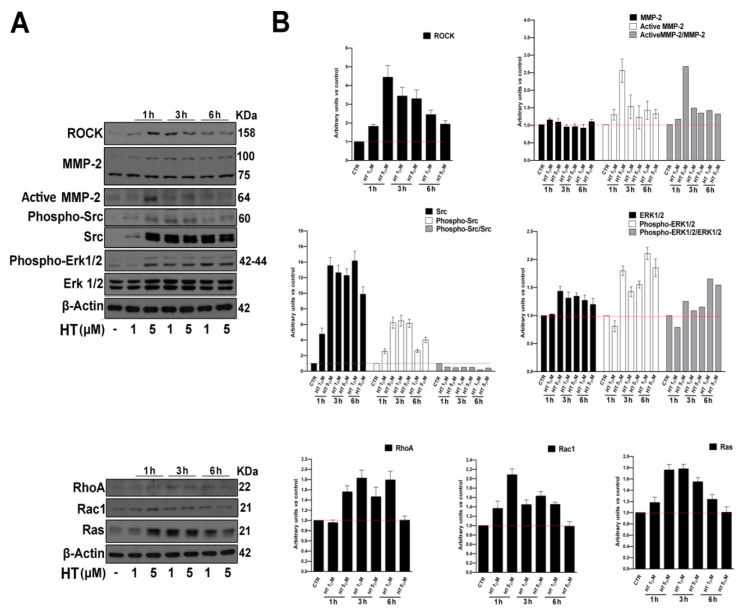
HT induces migration proteins expression in HUVEC cells. (**A**) Western blot analysis of ROCK, MMP-2, Phospho-Src, Src, Phospho Erk1/2, Erk1/2, RhoA, Rac1 and Ras in whole cell extracts from HUVECs treated for 1 h, 3 h and 6 h with HT at the indicated concentrations. β-Actin was used as control of protein loading. The panel shows a representative Western blot of three different experiments with similar results. (**B**) Histograms represent mean (±SD) in densitometry units of scanned immunoblots from three different experiments.

As is well-known, several factors are involved in the regulation of endothelial cell migration and angiogenesis, and it is crucial for the activation of signaling pathways that converge on cytoskeletal remodeling [23]. In order to establish the mechanism at the basis of HT stimulation of the migration process, we determined the expression of fundamental proteins involved in migration by western blot. To this end, we treated cells with HT at both concentrations (1 µM and 5 µM) for increasing time points (1 h, 3 h and 6 h), as shown in Figure 3A,B. We observed an upregulated expression of proteins that are implicated in cell adhesion, cytoskeletal dynamics and migration such as proto-oncogene tyrosine-protein kinase Src (Src), rho-associated protein kinase (ROCK), extracellular regulated protein kinases (ERK), ras homolog family member A (RhoA), ras-related C3 botulinum toxin substrate 1 (Rac1) and proto-oncogene, GTPase (Ras) [24,25,26,27], but also the activation of matrix metalloproteinase-2 (MMP-2), which is required for the degradation of the extracellular matrix and is involved in angiogenesis [28].

### 2.4. HT Induces Capillary Network Formation

**Figure 4:**
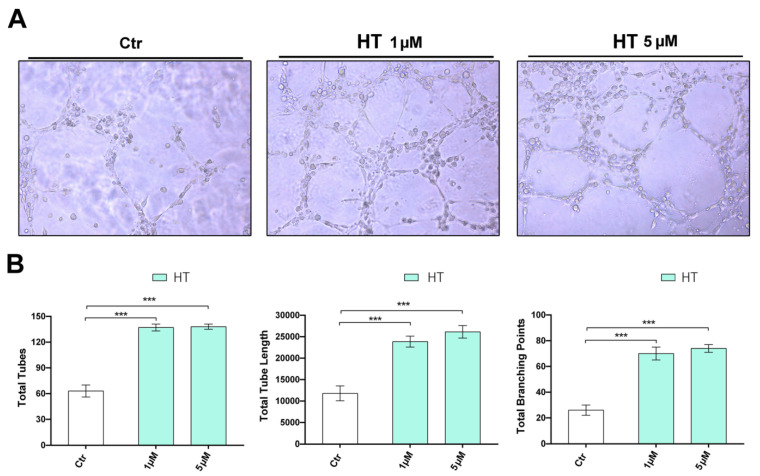
Effect of HT on tube formation. Pro-angiogenic benefit of HT on HUVECs tube formation was examined using a Matrigel assay. Tubular structures were photographed at 100 × magnification (**A**), and total tubes, the total tube length and total branching points were measured (**B**). Results are shown as mean (±SD) (2-way ANOVA, *** *p* < 0.001).

We next tested HT for its effect on the morphologic differentiation of endothelial cells into tube-like structures, a fundamental step for the angiogenesis process. To replicate in vitro the conditions that best mimic the *in vivo* microenvironment, which allows the differentiation of the capillaries, we plated endothelial cells into a Matrigel coat and carried out a 2-dimensional tube-formation assay. The angiogenic response was assessed by quantification of the capillary network that was formed after 6 h. HT, at the concentrations tested, promoted HUVECs tube formation, increasing both the number of total tubes, the overall tube length and the branching points (Figure 4A,B).

### 2.5. HT Induces the Expression of Angiogenesis Key Proteins

**Figure 5:**
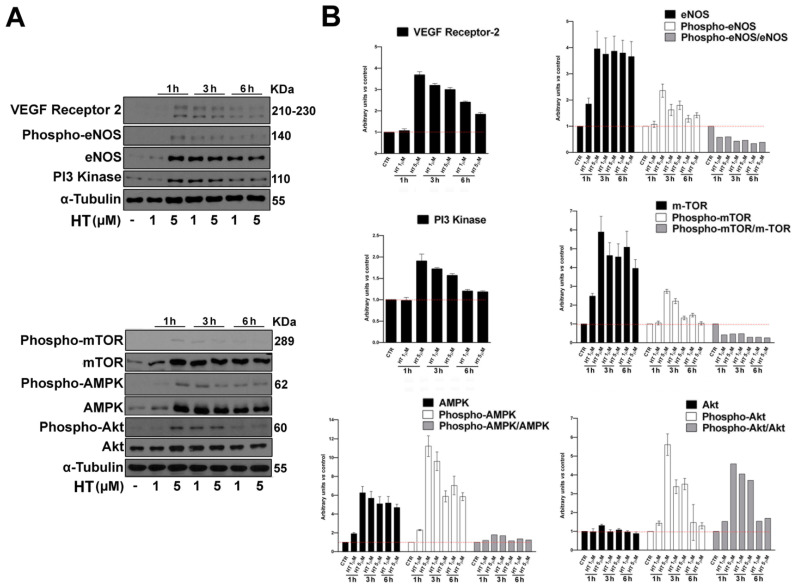
HT induces angiogenesis proteins expression in HUVEC cells. (**A**) Western blot analysis of VEGF Receptor-2, Phosho-eNOS, eNOS, PI3-Kinase, Phospho-mTOR, m-TOR, Phospho-AMPK, AMPK, Phospho-Akt and Akt in whole cell extracts from HUVECs cultured for 1 h, 3 h and 6 h in the presence of the indicated concentrations of HT. α-Tubulin was used as control of protein loading. The panel shows a representative Western blot of three different experiments performed with similar results. (**B**) Histograms represent mean (±SD) in densitometry units of scanned immunoblots from three different experiments.

To establish the mechanism at the basis of HT stimulation of the angiogenic process, we analyzed by western blot the expression of several proteins involved in the process, from angiogenic growth factors’ signaling, to proliferation and organization into new stable vessels. To this end, we treated cells with HT 1 and 5 µM for increasing time points (1 h, 3 h and 6 h), as shown in Figure 5A,B. We observed an upregulation in the expression and activation of the vascular endothelial growth factor (VEGF) receptor-2, eNOS, PI3-Kinase, m-TOR, AMPK and Akt, which are all involved in the formation and maturity of new capillary tubes [29]. It is well-recognized that eNOS plays a central role in endothelial cells, and that its activation is elicited by PI3K/AKT/mTOR pathway stimulation, which is involved in numerous cellular functions underlying angiogenesis, including proliferation, adhesion, migration and invasion, together with interconnected key angiogenic signal stimulators downstream VEGFR.

## 3. Discussion

The biological relevance of 3-Hydroxytyrosol (HT)—a phenolic alcohol found in olive oil, and the principal fat source in the Mediterranean diet—has been widely reported in scientific literature, thanks to its antioxidant, anti-inflammatory, anti-platelet aggregation and anti-atherogenic properties both in vitro and in animal models, which underscore its preventive and pharmacological potential [5,6].

In particular, the cardioprotective properties of olive oil have been assessed in numerous clinical studies. The Eurolive trial was one of the first to report the health benefits of daily extra-virgin olive oil consumption [30]. More recent trials have clearly demonstrated the association of a daily olive oil consumption within the Mediterranean diet pattern to a reduced incidence of chronic diseases, such as cardiovascular, metabolic, inflammatory disorders and cancer [31,32,33]. Only two studies have been conducted with direct HT supplementation. A daily intake of 5 mg of HT has been reported to protect low-density lipoproteins (LDL) from oxidation in the blood, while beneficial effects on dyslipidemia are still controversial, even if a recent reduction of LDL-cholesterol has been observed with pure HT administration associated with increased Vitamin C levels [34,35]. HT supplementation is also able to prevent neurodegenerative diseases, overall improving cognitive functions, mainly thanks to its anti-inflammatory properties and the overall improvement of vascular function [36,37]. Despite its success in preventing cardiovascular diseases, the therapeutic potential of HT for treating ischemic diseases is yet to be investigated in detail, especially with regards to ischemic heart disease, which remains a major challenge for humans. Therapeutic angiogenesis, which stimulates the growth of novel blood vessels from pre-existing ones and can restore blood flow to ischemic tissues, thus improving myocardial function, is certainly one of the most promising strategies to treat cardiovascular ischemic diseases. Several approaches are being pre-clinically studied or are already clinically employed to stimulate therapeutic angiogenesis, including the direct delivery of angiogenic growth factors or stem-cell based therapy. One of the major limitations found in the clinic compared to preclinical models is represented by the different recovery capacity in the vascularization of animal models compared to patients, where the increase in the capillary bed may not be enough to guarantee the recovery of hard-hit or necrotic ischemic tissues. In the last years, great advances have been made in a more efficient transfer of the most promising preclinical results to the clinic. Recent progress has been made utilizing polymeric biomaterials for drugs, growth factors or even cell delivery. Ultrasound targeted microbubble destruction has been proposed as a non-invasive gene therapy technique for clinical angiogenesis stimulation [38,39]. However, a combined therapy approach, targeting multiple components of angiogenesis with phytochemicals, might also be promising to achieve an effective stimulation of angiogenesis. For this reason, given its reported positive properties on cardiovascular function, in the present study we assessed the effect of HT on human umbilical vein endothelial cells as an in vitro angiogenesis model.

Several signaling events are involved in the regulation of endothelial cell migration, extension and contraction of cytoskeleton and angiogenesis [22,23]. We found that HT influences key molecules underscoring cytoskeletal dynamics fundamental for the angiogenic process. We have shown that HT activates and induces the expression of proto-oncogene tyrosine-protein kinase Src (Src), which, together with a ras homolog family member A (RhoA)/rho-associated protein kinase (ROCK) pathway, plays an important role in endothelial cell migration and proper tubulogenesis [23,24,25,26,27]. Angiogenesis requires the degradation of the extracellular matrix, along with the proliferation and migration of endothelial cells and synthesis of new matrix components. We found that the migration rate increase was supported by the activation of the matrix metalloproteinase-2 (MMP-2), which is involved both in normal tissue remodeling and angiogenesis [28]. The biological effects of olive oil polyphenols, including HT, on angiogenesis are controversial in the available literature [40,41,42,43,44]. Indeed, it has been shown that at high concentrations, HT is able to be anti-angiogenic in vitro, as well as in animal models of angiogenesis-dependent pathologies with a strong inflammatory component as rheumatoid arthritis [40,41,42,43,45,46,47]. The effect mainly relies on matrix metallopeptidase-2 (MMP-2), matrix metallopeptidase-9 (MMP-9), cyclooxygenase-2 (COX-2) and vascular endothelial growth factor receptor 2 (VEGF-R2) inhibition upon phorbol myristate acetate (PMA) proinflammatory stimulation [43]. As already pointed out for resveratrol, HT could have opposite effects on angiogenesis, depending on its concentration, stimulating the process at lower concentrations (<10 μM) and inhibiting it at higher ones (high micromolar-millimolar range). Such high concentrations cannot be achieved after the ingestion of olive oil, since polyphenols’ bioavailability is in the range of 0.1–1 μM [48]. Based on in vitro data, the prevalent biological effect of dietary polyphenols should be pro-angiogenic. Increasing adsorption through HT supplements could lead to higher blood levels, which might consequently inhibit angiogenesis in angiogenesis-dependent pathologies like cancer and atherosclerosis. Noteworthy, observational data from the PREDIMED clinical trial reported a lower incidence of cancer in long-term olive oil consumers, thanks to chemo-preventive properties mostly independent from its antioxidant effects [49]. Moreover, the biphasic effect of HT on angiogenesis seems to be highly dependent on the inflammatory context. According to the observed protective effects of HT on vascular functions in both cardiovascular and neurodegenerative pathologies, it has been observed that HT promotes cytoprotection and wound healing of vascular endothelial cells, and inhibits ROS-induced cell injury through Nrf2/HO-1 and FOXO3a/catalase pathways [50,51]. Moreover, HT prevents vascular endothelial dysfunction though increasing nitric oxide production and inhibiting TNFα-induced NFκB [52]. We confirmed in our model an upregulation of endothelial nitric oxide synthase (eNOS). It is well-recognized that eNOS plays a central role in endothelial function, and its impaired activation/expression results in reduced vasodilation, endothelial cell migration and angiogenesis in vitro [53,54,55]. The activation of eNOS is elicited by PI3K/AKT/mTOR pathway stimulation, which is involved in numerous cellular functions underlying angiogenesis, including proliferation, adhesion, migration and invasion, together with interconnected key angiogenic signal stimulators downstream VEGFR cascade as ERK and Ras. All these angiogenesis markers, fundamental for angiogenesis stimulation in wound healing, chronic inflammation and ischemic diseases, are activated by HT in HUVEC cells [53,54,55,56,57,58,59,60]. 

Our in vitro results revealed that HT promotes angiogenesis, providing evidence that it may be an effective therapeutic candidate for mitigating ischemic injury. Again, considering that oxidative stress plays a crucial role in the pathogenesis of cardiac diseases, and that HT can also regulate ROS levels, through both direct scavenging properties and indirect antioxidant efficacy, as shown by previous studies [16,61,62], the additional capability to protect vascular function and induce the angiogenic process in a pathological context configures it as a potential new therapeutic agent for ischemic diseases. “Overall, the relevance of our results on angiogenesis forms the preliminary basis of experiments to be confirmed ex vivo and in vivo”. With regards to the achievement of effective pharmacological doses of HT by taking olive oil, it is difficult to predict as the concentration and type of bioactive olive oil components is highly dependent on a quantity of agricultural factors, such as the cultivar and the pressing procedure. Furthermore, it is very difficult to standardize the quantification procedure of HT [49]. Nevertheless, a daily assumption of olive oil has well-documented health benefits, and HT supplementation has received European Food Safety Authority (EFSA) health claim approval, being reported to be safe, well-tolerated and contributing to the protection of LDL from oxidation [17,63]. So, a carefully designed diet including high-quality extra-virgin olive oil could be a rational basis to provide a safe and widely available strategy for cardiovascular prevention in healthy subjects, or in patients at higher risk of cardiovascular disease.

## 4. Materials and Methods

### 4.1. Chemicals and Materials

3-Hydroxytyrosol (HT) was purchased from Sigma-Aldrich Inc. (St Luis, MO, USA), solubilized in dimethyl sulfoxide (DMSO) (< 0.001% in our assays) and added to cell cultures at the reported concentrations. Matrigel-Matrix was purchased from BD Biosciences (Franklin Lakes, NJ, USA). Western blot analysis was used for the following antibodies: mouse monoclonal antihuman α-Tubulin; rabbit monoclonal anti-human phospho-mTOR; rabbit monoclonal anti-human mTOR; rabbit monoclonal anti-human phospho-AMPK; rabbit monoclonal anti-human AMPK; rabbit monoclonal anti-human Phospho-Akt (p-Akt; Ser473); rabbit monoclonal anti-human phospho-p44/42 MAPK (p-Erk1/2; Thr202/Tyr204); rabbit monoclonal VEGF Receptor-2; rabbit monoclonal anti-human p44/42 MAPK (Erk1/2); rabbit monoclonal anti-human Akt; rabbit monoclonal Phospho-eNOS; mouse monoclonal eNOS; rabbit monoclonal PI3-Kinase; and rabbit monoclonal anti-human rho-associated protein kinase (ROCK) (Cell Signaling Technology, Danvers, MA, USA). Mouse monoclonal matrix metalloproteinase-2 (MMP-2), mouse monoclonal Rac1, rabbit polyclonal anti-human RhoA, rabbit monoclonal anti-human Ras, rabbit polyclonal anti-human phosphor-Src (phospho Y418), rabbit polyclonal anti-human Src and rabbit polyclonal anti-human β-Actin were purchased from Abcam (Cambridge, UK). Secondary HRP-linked goat anti-mouse or goat anti-rabbit IgG were obtained from Cell Signaling Technology (Danvers, MA, USA). 

### 4.2. Cells

Human umbilical vein/vascular endothelium cells (HUV-EC-C [HUVEC]) were grown in a Vascular Cell Basal Medium (ATCC^®^, Manassas, VA, USA), supplemented with Microvascular Endothelial Cell Growth (ATCC^®^, Manassas, VA, USA). HUVEC cells were kindly provided by Prof. Carmine Vecchione (Department of Medicine, Surgery and Dentistry ‘Scuola Medica Salernitana’, University of Salerno, Baronissi, Salerno, Italy).

### 4.3. Determination of Cell Viability by MTT Assay

HUVEC cells (7 × 10^3^/well) were cultured into 96-well plates for 24 h before the addition of HT at the indicated concentrations, and cultured for an additional 24 h or 48 h at 37 °C. The reduction of the MTT (3-(4, 5-dimethylthiazolyl-2)-2, 5-diphenyltetrazolium bromide) tetrazolium salts assay was employed to examine cells’ viability, as described in detail elsewhere [64]. All experiments were performed in triplicate, and the relative cell viability was expressed as a percentage in comparison with the untreated control cells.

### 4.4. Determination of Cell Proliferation by BrdU Assay

HUVEC cells (7 × 10^3^/well), were cultured into 96-well plates for 24 h before the addition of HT at the indicated concentrations and cultured for additional 24–48 h at 37 °C. Cell proliferation was evaluated by measuring BrdU incorporation into DNA (BrdU colorimetric assay kit; Roche Applied Science, South San Francisco, CA, USA), by an ELISA plate reader (Thermo Scientific, Waltham, MA, USA) at 450 nm, as described in detail elsewhere [65]. In all experiments, performed in triplicate, the relative cell growth was expressed as percentage in comparison with untreated 24 h control cells (100%).

### 4.5. Scratch Wound Healing Assay

HUVEC cells were plated in 6-well plates at a density of 10 × 10^3^ cells/well. When the confluent cells formed a homogeneous carpet, a vertical wound in the wells was performed using a 200 µL tip. After a careful wash to eliminate the cells detached from the plate, the culture medium containing HT at the indicated concentrations was added to the wells. The wound area was recorded instantly and after 6 h through microscope analysis, and quantified by Wimasis Image Analysis software (Onimagin Technologies Spa, Cordoba, Spain).

### 4.6. Boyden Chamber Cell Migration Assay

Cell migration was performed by Boyden chamber trans-well system (Falcon). Cell suspensions (300 µL, 30.000 cells) were added to a 900 µL medium and plated in pre-coated cell culture inserts with 8 µm pore size membranes placed into 12-well plates. Cells were treated overnight with HT. At the end of the experiment, inserts were washed three times with phosphate-buffered saline (PBS), and cells on the underside of the membrane were fixed with cold methanol for 15 min, and then stained with Giemsa solution for 15 min. The membranes were washed, removed and mounted on glass slides and cells were counted. The assays were performed in triplicate wells for each condition, and each experiment was repeated at least three times. 

### 4.7. Capillary-Like Tube Formation on Gel

Prechilled 48-well plates were coated with Matrigel, which was allowed to polymerize for 30 min at 37 °C. HUVEC cells were seeded (1 × 10^4^ cells/well) in 250 μL of complete medium with HT. After 6 h, a capillary-like tube formation was examined and photographed by an inverted phase microscope. The number of tubes, network intersections and the total tube length were quantified by Wimasis Image Analysis software, (Onimagin Technologies Spa, Cordoba, Spain). Each experiment was repeated at least three times.

### 4.8. Western Blot Analysis

Cells were grown in p100 tissue culture plates at a density of 2 × 10^4^ cells/cm^2^ for 24 h. Cells were then incubated with HT, as indicated. After incubation, cells were washed with PBS, harvested and lysed in ice-cold radioimmunoprecipitation assay (RIPA) lysis buffer (50 mM Tris-HCl, 150 mM NaCl, 0.5% Triton X-100, 0.5% deoxycholic acid, 10 mg/mL leupeptin, 2 mM phenylmethylsulfonyl fluoride and 10 mg/mL aprotinin), and then assayed for Western Blot by the procedure, which is described in detail elsewhere [66].

### 4.9. Statistical Analysis

Statistical analysis was performed by GraphPad prism 6.0 software for Windows (GraphPad software, San Diego, CA, USA). For each type of assay, data obtained from multiple experiments have been calculated as mean ± (SD) and analyzed for statistical significance using the 2-tailed Student t-test, for independent groups, or 2-way ANOVA followed by Tukey post-hoc correction for multiple comparisons. *P* values less than 0.05 were considered significant. * *p* < 0.05, ** *p* < 0.01 and *** *p* < 0.001.

## 5. Conclusions

We provide the first demonstration of how low 3-Hydroxytyrosol (HT) concentrations, which reach the bloodstream after olive oil consumption, are able to promote endothelial cell migration, influencing signal transduction and the subsequent formation and maturity of new capillary tubes. Our data argue in favor of a great pharma-nutritional potential for HT through multi-targeted actions, including the stimulation of angiogenesis, corroborating its cardio-preventive properties. A pre-clinical study, using mesenteric arteries as an ex-vivo model, is currently underway to evaluate the role of differential doses of HT on vascular function.
